# The Development and Validation of a Dehumanization Measure Within Romantic Relationships

**DOI:** 10.3389/fpsyg.2019.02754

**Published:** 2019-12-06

**Authors:** Bengianni Pizzirani, Gery C. Karantzas, Ellie R. Mullins

**Affiliations:** ^1^Science of Adult Relationships, School of Psychology, Faculty of Health, Deakin University, Melbourne, VIC, Australia; ^2^School of Public Health and Preventative Medicine, Faculty of Medicine, Nursing and Health Sciences, Monash University, Melbourne, VIC, Australia

**Keywords:** dehumanization, interpersonal relationships, confirmatory factor analysis, measurement, abuse, maltreatment

## Abstract

Despite the emergence of research into interpersonal dehumanization, there has been little by way of empirical investigation of the phenomenon within the context of romantic relationships. To address this, we introduce and validate the Dehumanization in Romantic Relationships Scale (DIRRS), a self-report measure of dehumanization perpetration and targeting within close relationships. In Study 1 (*N* = 1251, *M age* = 25.35, *SD* = 6.03), confirmatory factor analysis revealed that the dimensionality of interpersonal dehumanization may be more nuanced than first thought. Specifically, a four factor first-order structure [comprised of factors that relate to denials of human uniqueness (i.e., immature and unrefined) and human nature (i.e., exploitable and emotionless) was found to be the best fit to the data]. These results were replicated on a different sample in Study 2 (*N* = 847, *M age* = 23.40, *SD* = 6.43)—in addition to the assessment of criterion-related validity. Study 3 (*N* = 328, *M age* = 23.40, *SD* = 6.43) cross-validated the criterion-related validity reported in Study 2, and in addition, highlights that dehumanization is also associated with emotional and physical abuse. This research extends theory on interpersonal dehumanization and provides an empirically validated measure to reliably assess the occurrence of dehumanization within romantic relationships.

## Introduction

Dehumanization—defined as the denial of uniquely or fundamentally human characteristics to another—is considered to occur across a range of contexts including moral atrocities related to ethnic cleansing, inter-racial conflicts, as well as medical and organizational settings (Bain et al., 2014; Haslam and Stratemeyer, 2016). However, theoretical advancements over the past decade, suggest that dehumanization is not limited to intergroup conflicts or blatant acts of atrocity. Rather, dehumanization can take the form of implicit and commonplace behaviors that manifest within interpersonal contexts, but nonetheless, damage relational bonds (e.g., social ostracism and contempt; Bastian and Haslam, 2011; Bastian et al., 2014). For example, being socially ostracized or treated instrumentally (i.e., as if one’s value is based solely on what they have to offer another) by a friend.

Despite the emergence of research into interpersonal dehumanization, there has been little by way of empirical investigation of dehumanization within the context of romantic relationships. Nevertheless, many of the behaviors identified as negative and toxic within romantic relationships such as hostility, ridicule, and being controlling reflect dehumanization (Pizzirani and Karantzas, 2019). Specifically, such behaviors deny the target (i.e., the romantic partner) their sense of agency, or treats them as if they have no feelings or lack intelligence (Bastian and Haslam, 2011). Moreover, interpersonal dehumanization within close relationships is often characteristic of common and subtle maltreatments, such as feigning disinterest, acting in ways that may disrespect another, or minor exploitative acts such as requesting favors that see another treated as a means to an end (Bastian et al., 2014).

Through a series of studies, Bastian and Haslam (2011) found that targets of a range of common maltreatments experienced dehumanization. Such work demonstrates that the actions of close others—such as romantic partners—play an important hand in people’s experiences of dehumanization. Furthermore, acts of dehumanization within intimate contexts are likely to disrupt the positive functions of romantic relationships, which include the provision of support to encourage competence and personal growth within a partner (Feeney and Collins, 2014), and to meet a partner’s fundamental needs for comfort and security (Feeney, 2009).

Given the increasing interest in interpersonal dehumanization, it is surprising that there is little by way of dehumanization research within the context of intimate relationships. We argue that this dearth in research is for two reasons. Firstly, there exists no target or perpetrator assessment of dehumanization within romantic relationships. This is despite findings that demonstrate interpersonal dehumanization is most often perpetrated by people within one’s close social network, which includes romantic partners (Adams, 2014). Secondly, conceptual ambiguity surrounds the dimensionality of interpersonal dehumanization (and dehumanization more generally). For example, some research suggests that interpersonal dehumanization is best conceptualized as a two-dimensional construct (i.e., the denial of human nature and the denial of human uniqueness; Bastian and Haslam, 2010, 2011), while other studies suggest that interpersonal dehumanization may be unidimensional (Bastian et al., 2012a, b, 2014). To address these limitations, we report on three studies in which we developed and validated a measure to assess dehumanization (from both target and perpetrator perspectives) within romantic relationships, as well as determine the dimensional structure of the construct.

Traditionally, dehumanization was viewed as an extreme phenomenon that took place within the contexts of mass violence (i.e., mass shootings), intense conflict (i.e., the Syrian War, Kelman, 1976) and moral atrocities (i.e., the genocide against the Tutsi in Rwanda, Bandura, 1999). However, since this early work, dehumanization has been acknowledged to be a commonly experienced phenomenon within interpersonal contexts (such as couple, family, and peer relationships) that can include subtle (e.g., Leyens et al., 2000) and seemingly innocuous maltreatments such as being treated with condescension, contempt, or anger (e.g., Bastian and Haslam, 2011).

Up until the last decade, dehumanization was described as a unidimensional construct such that it reflected denying individuals the human qualities that separate them from animals (e.g., Leyens et al., 2000). In an attempt to provide a more comprehensive conceptualization of dehumanization, Haslam (2006) proposed a model of dehumanization comprising of two-dimensions. According to Haslam, dehumanization can entail behaviors beyond likening an individual or group to an animal. Haslam proposed that people also tend to engage in dehumanization that likens an individual or group to inanimate objects, such as machines and robots. To this end, these two distinct denials of humanness yield two-dimensions of dehumanization.

According to this two-dimensional perspective (Haslam, 2006), dehumanization can involve the denial of uniquely human characteristics and characteristics reflective of human nature. When denied uniquely human qualities (also referred to as ‘animalistic’ dehumanization), people are likened to animals and seen as primitive, inferior, irrational, childlike, or unintelligent. Specifically, people are denied attributes such as self-control, civility, competency, social refinement, agency (e.g., the ability to plan and think for oneself) and maturity (Haslam et al., 2013). When denied human nature qualities (also referred to as ‘mechanistic’ dehumanization), people are likened to objects and machines and seen as superficial, preprogramed, cold, or lacking emotion (Bastian and Haslam, 2011; Haslam et al., 2013). This form of dehumanization involves denying people attributes such as emotionality, cognitive flexibility, curiosity, and interpersonal warmth (Haslam et al., 2013).

The two-dimensional conceptualization of dehumanization (Haslam, 2006) has received considerable support from a number of studies (for review see Haslam, 2015). However, recent research again suggests a unidimensional conceptualization of dehumanization may be more appropriate within interpersonal contexts. For example, across four studies Bastian et al. (2012a) identified a unidimensional factor solution for dehumanization^1^ (as opposed to a two-factor solution), suggesting that the denial of human nature and human uniqueness may be subsumed under a single dimension. Thus, although a number of studies support the distinction between human uniqueness and human nature (see Haslam, 2015), recent research has brought this conceptualization into question, especially within the interpersonal context (e.g., Bastian et al., 2012b).

The issues regarding the dimensionality of interpersonal dehumanization may, in part, be a function of the history of the measurement of dehumanization (which has stemmed largely from intergroup research of the phenomenon). Specifically, assessment has largely involved either single item measures or multi-item perceptions of humanness traits. Single item measures, for instance, assess a particular group’s evolutionary progress on a question depicting the ascent of man (Kteily et al., 2015). However, this measure only assesses animalistic dehumanization (e.g., perceiving others as ‘savage’ and ‘barbaric’) and not the denial of human nature.

Self-report perceptions of humanness involve rating a set of personality-like characteristics as reflecting either human uniqueness or human nature (e.g., Haslam et al., 2005; Loughnan and Haslam, 2006). Derivatives of these measures assess the extent to which these traits are perceived within the self, and/or ascribed to others (e.g., Bain et al., 2009; Bastian et al., 2012b). Other self-report measures assess perceptions of having experienced dehumanization (e.g., Bastian and Haslam, 2010, 2011). However, while these measures have been used to explicitly assess denials of human uniqueness and human nature, recent findings suggest either two semi-independent factors or two highly correlated factors with researchers collapsing across these dimensions to yield a single dehumanization factor (see Bastian et al., 2012a, b). In addition, these measures suffer from three further limitations when assessing dehumanization within the context of close relationships.

Firstly, the vast majority of existing self-report assessments focus on ascribing or evaluating human characteristics rather than explicitly measuring behaviors that reflect dehumanization—that is—the *actual treatment* of someone as less than human. Within close relationships, the interpersonal processes that govern relationship functioning often entail a behavioral component [e.g., conflict patterns (Eldridge and Christensen, 2002), the provision of social support (Feeney and Collins, 2014), and the demonstration of intimacy (Laurenceau et al., 1998)]. To this end, it would be remiss of dehumanization assessments to not capture behavioral manifestations of the construct in close relationships.

Secondly, measures to date (largely because of the field’s focus on the intergroup context) reflect dehumanization attitudes or perceptions toward people or groups in general. However, within the context of close relationships, the fidelity of assessment can be higher and the predictive validity of the measure greater, when the assessment focuses on a specific individual (e.g., romantic partner, peer, or parent; Fraley et al., 2011).

Finally, studies into dehumanization tend to focus, and therefore assess, perceptions or attitudes regarding being either the perpetrator of dehumanization or the target of dehumanization, but not both (e.g., Haslam et al., 2005; Kteily et al., 2015). Nevertheless, within an interpersonal context such as romantic relationships, dehumanization should be considered dyadic in nature. In this way a person’s experiences of dehumanization will entail either treating one’s partner as less than human (i.e., perpetration) and/or being the target of dehumanization enacted by one’s partner. Hence, a comprehensive measurement of the construct within the context of intimate relationships should involve the assessment of dehumanization from both perspectives.

In light of these limitations, it is necessary to develop a psychometrically sound measure of romantic relationship dehumanization. Moreover, the development of such a measure can help relationship scholars better understand the role of dehumanization in the manifestation of aversive relationship behaviors [e.g., cycles of violence (Bastian et al., 2014) or intimate partner aggression].

The first aim of this paper is to determine the dimensionality of dehumanization within romantic relationships by comparing the two most common conceptualizations of interpersonal dehumanization proposed within the literature—the unidimensional structure (as reported by Bastian et al., 2012a, b) and the two-dimensional structure (reflecting Haslam, 2006 dual model of dehumanization). The second aim is to develop and psychometrically assess (i.e., construct validity and criterion-related validity) a measure of romantic relationship dehumanization that captures both perpetrator and target perspectives.

The development and psychometric evaluation of this new measure, titled the ‘Dehumanization in Romantic Relationships Scale’ (DIRRS), is reported across three Studies. Study 1 reports on the initial development and construct validity of the DIRRS and includes a comparison of the proposed unidimensional and two-dimensional structures. Study 2 reports on a cross-validation of the DIRRS and extends the psychometric evaluation to criterion-related validity (i.e., concurrent validity). Specifically, a series of measures pertaining to relationship functioning such as relationship quality, patterns of communication and negative interactions, providing care for one’s partner, and maintaining positive partner regard are used to determine concurrent validity. Study 3 attempts to replicate the criterion-related validity findings of Study 2 but extends on this to explore the associations between dehumanization and overt emotional and physical abuse within romantic relationships.

## Study 1

Study 1 reports on the development of the DIRRS and the psychometric evaluation of the measure (i.e., construct validation), thus identifying the optimal factor structure and determining the internal consistency (i.e., reliability) of the scales that constitute the DIRRS. Given the construct of interpersonal dehumanization has been articulated to constitute either a single dimension or two-dimensions it was important to: (1) develop an item pool that captured the full breadth of the construct across these differing conceptualizations, and, (2) statistically model and compare the unidimensional and two-dimensional conceptualizations of interpersonal dehumanization.

### Overview of Item Pool Development

The development of the DIRRS involved deriving items that captured the definitional properties of both the unidimensional and two-dimensional conceptualizations of interpersonal dehumanization, as well as the full range of hurtful relationship behaviors suggested in the literature to represent interpersonal denials of humanness (for review see Pizzirani and Karantzas, 2019). A literature search was also conducted to identify past measures within the general field of dehumanization research.

Using the definitional features of human uniqueness (i.e., intelligence, self-control, civility, competency, social refinement, and maturity) and human nature (i.e., the ability to experience and express emotions, cognitive flexibility, and interpersonal warmth), the relational denials of humanness identified by Pizzirani and Karantzas (2019), and drawing on Bastian and Haslam (2011) measure of dehumanization^2^, an initial pool of 30 items were developed that could be equally framed to capture both perpetrator and target perspectives of romantic dehumanization.

### Modeling the Dimensional Structure of Romantic Relationship Dehumanization

**FIGURE 1:**
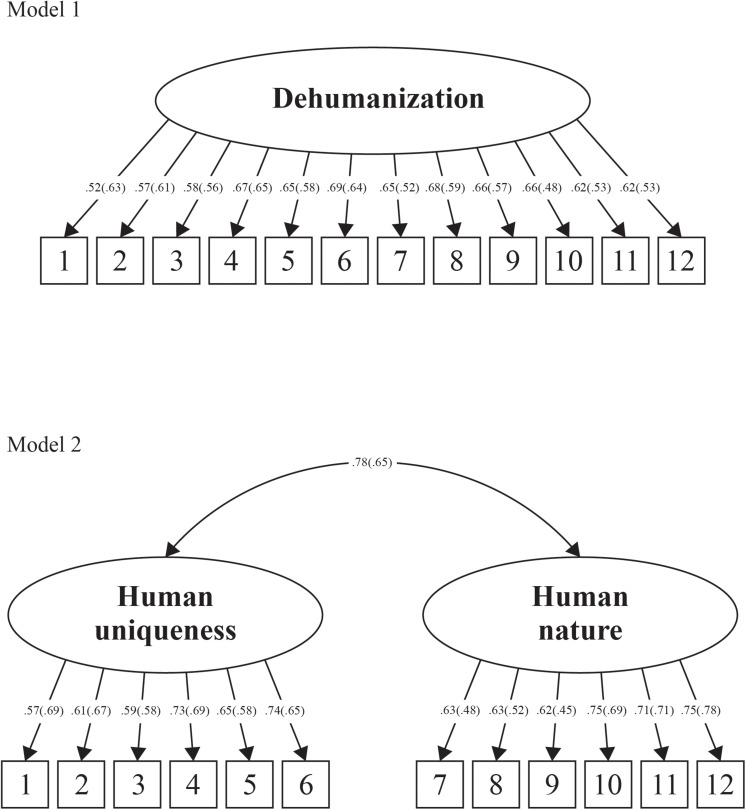
Alternative conceptualizations of dehumanization. Standardized factor loadings and covariances for perpetration items are presented in parentheses.

The construct validation approach for Study 1 involved the *a priori* modeling and comparison of two factor structures. The first of these factor structures (Figure 1, Model 1) depicted dehumanization as a unidimensional construct, such that all items were modeled to load onto a global dehumanization factor. This factor structure draws on recent empirical evidence (as well as early theorizing on the concept of dehumanization) to suggest that the construct is best represented as unitary in nature (e.g., Bastian et al., 2012a, b, 2014). The second factor structure (Figure 1, Model 2) depicted dehumanization as a two-dimensional construct constituting the denial of human uniqueness and the denial of human nature. This factor structure represents Haslam (2006) dual model of dehumanization. Given the suggestion that interpersonal dehumanization may reflect quite nuanced manifestations of dehumanization (e.g., Pizzirani and Karantzas, 2019) we predicted that modeling the two-dimensional conceptualization on the DIRRS would provide better fit to the data than the unidimensional conceptualization.

### Method

#### Participants

The sample consisted of 1251 participants (39.2% men, 60.1% women, 0.6% gender not specified) recruited online from social networking sites and online forums (e.g., Facebook and Reddit). The sample consisted of participants from the United States (54.8%), Canada (10.6%), Australia (8.6%), and the United Kingdom (8.2%). Participants ranged in age from 18 to 63 years (*M* = 25.35, *SD* = 6.03) and were all in a current romantic relationship (*M* relationship length = 3.33 years, *SD* = 3.83). Relationship status of the sample comprised of individuals who were steadily dating (49.9%), cohabiting (25.7%), engaged (8.1%), and married (17.4%).

#### Materials and Procedure

This study received ethics approval from the Deakin University’s Human Ethics Committee. The DIRRS is presented in Appendix A. An initial pool of 30 items was created and items were framed to capture both perpetrator and target perspectives of romantic relationship dehumanization. Two experts then independently reviewed and rated the face-validity of all 30 items. A face-validity approach was used to ensure that the generated items were judged to appropriately assess the intended construct (e.g., Murphy and Davidshofer, 1988; Nunnally and Bernstein, 1994; Hardesty and Bearden, 2004) and to limit the exclusive reliance on empirical data reduction approaches such as Exploratory Factor Analysis.

The face-validation of items was based on three criteria. These were the extent to which each item: (1) captured the unidimensional and two-dimensional conceptualizations of dehumanization, (2) reflected dehumanization within romantic relationships, and (3) applied to both perpetrator and target perspectives of dehumanization. The two experts were also instructed to limit the measure’s length to ensure that it could be completed in a timely manner. To guide the number of items to be selected, the independent reviewers were asked to select up to six items reflective of the denial of human uniqueness and human nature. The decision to limit the item selection in this way was on the basis of Classical Test Theory and latent variable modeling approaches to measurement development (e.g., Harvey et al., 1985; Hinkin, 1995, 1998; Marsh et al., 2010; Brown, 2015).

The two independent reviewers demonstrated high agreement (Cohen’s Kappa = 91.2%) across the selection of 12 items from the original item pool. These 12 items were categorized by both independent raters to reflect either the denial of human uniqueness (six items) or the denial of human nature (six items). Of the final 12 items selected by the expert judges, three were items that were retained from the Bastian and Haslam (2011) dehumanization measure, while nine reflected original items (see Appendix A). As shown in Appendix A, each item is worded in two forms to reflect assessment of both perpetrator and target perspectives. Thus, participants are required to rate the same 12 items *twice* – once to assess their tendency to perpetrate dehumanization, and once to assess their tendency to be the target of dehumanization. Each item is rated on a seven-point Likert scale ranging from 1 (*strongly disagree*) to 7 (*strongly agree*). Higher scores reflect greater dehumanization perpetration or targeting.

Participants were administered the DIRRS by way of an anonymous online survey that also included a demographics questionnaire that entailed sex, age, ethnicity, relationship length, and relationship status. The online survey took approximately 10 min.

#### Data Analysis

The construct validity of the DIRRS was assessed using confirmatory factor analyses (CFA) with Maximum Likelihood Estimation (Muthèn and Kaplan, 1985). Assessment of model fit was based on the guidelines proposed by Hu and Bentler (1999). Therefore, in addition to evaluating the chi-square value (χ^2^_ML_), a model with a Comparative Fit Index (CFI) and Tucker Lewis Index (TLI) ≥ 0.95, Root Mean Square Error of Approximation (RMSEA) ≤ 0.05, and Standardized Root Mean Residual (SRMR) = 0.06 was indicative of good fit. Given that the secondary aim of this research was to determine the dimensional composition of interpersonal dehumanization, CFA was used to model two alternative factor structures – Model 1 evaluated the unidimensional conceptualization of dehumanization, while Model 2 evaluated the two-dimensional conceptualization. Because the newly developed measure included items that assessed dehumanization from both the perpetrator and target perspectives, separate CFAs were conducted for each set of items. In order to determine the best fitting structure between the two alternative models (i.e., unidimensional and two-dimensional structures) chi-square difference (Δχ^2^) tests of model fit were conducted. To safeguard against Type II error when identifying significant differences between models, a practical difference test (i.e., a TLI difference of 0.01) was employed.

### Results and Discussion

The unidimensional structure of dehumanization (see Figure 1, Model 1) demonstrated poor fit for both perpetration and target versions of the DIRRS (see Table 1). The two-dimensional structure (see Figure 1, Model 2) also demonstrated poor fit for the items constituting the perpetration of dehumanization and being the target of dehumanization (see Table 1). Factor loadings for all items (perpetration and target) across the two alternative models (i.e., Models 1 and 2) varied in magnitude from λ = 0.45 to λ = 0.78 (see Figure 1) and were all significant at *p* < 0.001. As presented in Table 1, comparison of the fit of perpetration and target items across Models 1 and 2 by way of chi-square differences tests revealed that the two-dimensional model was of significantly better fit to data for both sets of items compared to the unidimensional model.

**TABLE 1:** Study 1 Chi-square difference tests for perpetration and target versions of the DIRRS.

**Model**	**χ^2^**	**df**	**CFI**	**TLI**	**RMSEA**	**SRMR**	**Comparison**	**χ^2^Δ**	**df Δ**
**Perpetration**									
Model 1 (single factor)	1702.778	54	0.688	0.618	0.156	0.095	Model 1 and Model 3	1436.471^∗∗∗^	6
Model 2 (2 correlated factors)	1281.402	53	0.767	0.710	0.136	0.091	Model 2 and Model 3	1015.095^∗∗∗^	5
Model 3 (4 correlated factors)	266.307	48	0.959	0.943	0.060	0.044	–	–	–
Model 4 (4 first-order factors, 2 second-order factors)	289.491	49	0.954	0.939	0.063	0.049	Model 4 and Model 3	23.341^∗∗∗^	1
Model 5 (4 first-order factors, 1 second-order factor)	299.648	50	0.953	0.938	0.063	0.048	Model 5 and Model 3	33.341^∗∗∗^	2
**Target**									
Model 1 (single factor)	1788.304	54	0.741	0.683	0.160	0.086	Model 1 and Model 3	1438.818^∗∗∗^	6
Model 2 (2 correlated factors)	1509.947	53	0.782	0.729	0.148	0.085	Model 2 and Model 3	1160.461^∗∗∗^	5
Model 3 (4 correlated factors)	349.486	48	0.955	0.938	0.070	0.044	–	–	–
Model 4 (4 first-order factors, 2 second-order factors)	349.524	49	0.955	0.940	0.070	0.044	Model 4 and Model 3	0.038	1
Model 5 (4 first-order factors, 1 second-order factor)	349.611	50	0.955	0.941	0.069	0.044	Model 5 and Model 3	0.125	2

*χ^2^, Chi-square; df, degrees of freedom; CFI, Comparative Fit Index; TLI, Tucker Lewis Index; RMSEA, root mean square error of approximation; SRMR, standardized root mean residual. ^∗∗∗^*p* < 0.001.*

However, despite the significantly better fit of the two-dimensional model, the overall poor fit of this factor structure indicates model misspecification (e.g., Brown, 2015). In particular, a poor fitting factor structure can often signal that item variance needs to be accounted for through the modeling of additional factors (e.g., Brown, 2015; Kline, 2015). To this end, we endeavored to derive a *post hoc* multifactorial structure that would: (1) better account for item variance, and (2) advance on prior theory regarding the dimensionality of dehumanization.

*Post hoc* re-examination of the items for human uniqueness and human nature suggested that each subscale of the DIRRS may in fact contain subthemes suggestive of a four factor solution for the perpetrator and target items respectively. While a four factor conceptualization of dehumanization is indeed novel, a closer inspection of the literature indicates an implicit set of facets pertaining to human uniqueness and human nature that are related, but somewhat distinct. Specifically, these facets can be found within the vast majority of contemporary definitions of human nature and human uniqueness (e.g., Haslam, 2006, 2015; Bastian and Haslam, 2011; Haslam et al., 2013). For example, the denial of human uniqueness is described as perceiving the target as coarse and backward (i.e., lacking social refinement), or irrational, childlike and immature (i.e., lacking maturity and competence). The denial of human nature is often described as seeing the target as an object or machine (i.e., a means to an end) or as cold, inert, and lacking emotion (i.e., heartless).

Therefore, the denial of human uniqueness may reflect two subfactors with items (3 per factor) constituting a lack of immaturity and social refinement. The ‘*immature*’ factor represents the treatment of an individual as if they are a child and can’t manage on one’s own, whereas the ‘*unrefined*’ factor suggests that an individual is an embarrassment to one’s partner, and that one’s partner is ashamed of an individual’s lack of social status. Likewise, the denial of human nature also reflects two subfactors (three items per factor), namely, that the partner is exploitable and emotionless. The ‘*exploitable*’ factor represents being treated as a means to an end in which one’s worth is based on what they can offer another (i.e., they are a machine or robot-like), whereas the ‘*emotionless*’ factor suggests that one is heartless and unresponsive (i.e., lacking emotion).

**FIGURE 2:**
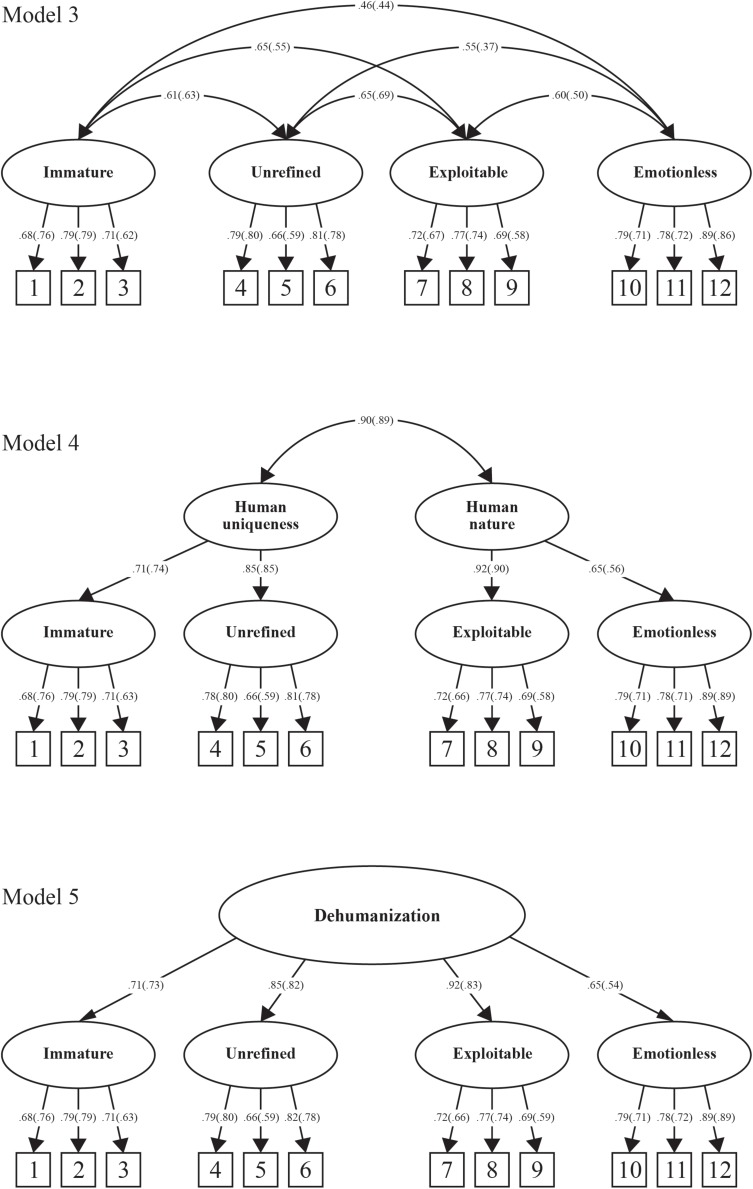
New conceptualizations of interpersonal dehumanization. Standardized factor loadings and covariances for perpetration items are presented in parentheses.

To determine the fit of this multifactorial structure, a four factor first-order structure with oblique rotation was evaluated (see Figure 2, Model 3). However, given that Model 3 reflected a multifactorial structure that was derived on the basis of items thought to capture the unidimensional and two-dimensional conceptualizations of dehumanization, two additional higher-order models were tested. Specifically, Model 4 (see Figure 2) represents a two factor higher-order model with four first-order factors. The two higher-order factors represent the dual model of dehumanization. Model 5 (see Figure 2) represents a single factor higher-order model with four first-order factors. The higher-order factor represents the unidimensional conceptualization of interpersonal dehumanization. That is, both higher-order models assume that the existing conceptualizations of dehumanization reported in the literature may reflect higher-order or global structures that are underpinned by more nuanced and specific lower-order facets. As in the case of Models 1 and 2, Models 3 to 5 were evaluated for model fit and comparison across models was conducted using chi-square difference tests.

The four factor first-order structure of dehumanization (Figure 2, Model 3) resulted in very good fit for both perpetration and target items (see Table 1). The two-dimensional higher order structure (Figure 2, Model 4) demonstrated good to very good fit for both perpetration and target items (see Table 1). The unidimensional higher-order structure (Figure 2, Model 5) also resulted in good to very good fit for both perpetration and target items (see Table 1). Across Models 3 to 5, the scale items demonstrated moderate to high loadings onto their respective factors (λ = 0.58–0.89, see Figure 2) and all loadings were significant (*p* < 0.001).

Chi-square difference tests comparing the fit between Models 3 through 5 with Model 2 (the best fitting model out of Models 1 and 2) were conducted across perpetration and target items. In relation to items assessing perpetration, Model 3 (i.e., the four factor first-order solution) demonstrated significantly better fit when compared to all other models (see Table 1). In relation to items assessing being the target of dehumanization, Models 3 through 5 were of significantly better fit compared with Model 2, however, the differences between Models 3, 4, and 5 (Δχ^2^ and ΔTLI) were not significant. That is, Models 3 to 5 were equivalent in terms of fit (see Table 1).

In summary, model comparisons revealed that the four factor first-order solution (Model 3) was the best fit to the data in relation to the perpetration items. Model 3 comprised factors that relate to denials of human uniqueness (i.e., immature and unrefined) and human nature (i.e., exploitable and emotionless). The four factor first-order model possesses good internal consistency across both perpetration (immature α = 0.76; unrefined α = 0.75; exploitable α = 0.71; emotionless α = 0.83) and target (immature α = 0.76; unrefined α = 0.78; exploitable α = 0.76; emotionless α = 0.85) items.

While the superior fit of Model 3 was apparent across all comparisons based on the perpetration items of the DIRRS, the findings were not as consistent for the target items. Specifically, Model 3 demonstrated significantly better fit than Models 1 and 2, however, was of equivalent fit when compared to Models 4 and 5 (i.e., the two factor and single factor higher-order models). While further confirmation of this new factor structure is required, what is apparent is that the dimensionality of the DIRRS (and interpersonal dehumanization more generally) may be more nuanced than first thought. That is, the two most common conceptualizations of dehumanization proposed within the literature—the unidimensional structure (as reported by Bastian et al., 2012a, b) and the two-dimensional structure (reflecting Haslam (2006) dual model of dehumanization)—may not best reflect the nature of interpersonal dehumanization, especially within romantic relationships.

## Study 2

The findings for the construct validation of the DIRRS in Study 1 resulted in a four factor first-order structure (i.e., Figure 2, Model 3) that had not been theorized *a priori*. Therefore, the central aim of Study 2 was to cross-validate this structure on an independent sample. In doing so, we again compared the fit of the four factor first-order structure to all other dehumanization models tested as part of Study 1 [i.e., Models 1, 2 (Figure 1), 4, and 5 (Figure 2)]. We also extend our psychometric evaluation of the DIRRS to a criterion validity analysis for both versions (target and perpetration) of the measure.

Given that dehumanization reflects people’s behavioral tendencies to treat another as less than human, it is highly probable that being the perpetrator and/or target of dehumanization is likely to be associated with a constellation of negative relationship behaviors and outcomes. For instance, dehumanization perpetration is characterized by treating someone as incompetent, stupid or as a means to end (Haslam, 2006), and is thus likely to be associated with engaging in other toxic and aversive relationship behaviors. These can include limiting a person’s agency and autonomy through controlling and intrusive behavior often manifested as caregiving or social support (e.g., Kunce and Shaver, 1994; Bolger and Amarel, 2007; Pizzirani and Karantzas, 2019). Similarly, targets of romantic relationship dehumanization are likely to report being treated insensitively, suffering ridicule, and hostility. Therefore, targets may also engage in reactive or defensive behaviors in response their partner’s dehumanization, such as engaging in destructive communication patterns (e.g., stonewalling or withdrawing).

Conversely, being either the perpetrator or target of dehumanization should attenuate people’s engagement in positive and constructive relationship behaviors while also lessening their positive appraisals of their partner and the relationship overall. For example, based on the perpetrator’s treatment of their romantic partner as less than human, we would expect the perpetrator to possess less positive regard for their partner as well as reductions in the perceived quality of their relationship. This is because the target is unlikely to be evaluated by the perpetrator as living up to what is expected of a partner (Fletcher et al., 1999). On the other hand, the target of dehumanization is also likely to view their partner and the relationship in a less positive light given that the behaviors that constitute dehumanization are likely to undermine the positive functions of romantic relationships in which the perpetrator may also be viewed as falling well short of the target’s ideas of how they should be treated within their romantic relationship (Fletcher et al., 1999; Simpson et al., 2001). Therefore, the second aim of Study 2 was to determine the concurrent validity of the DIRRS using a series of measures pertaining to relationship functioning as well as partner and relationship appraisals.

### Method

#### Participants

The sample consisted of 847 participants (37.9% men, 61.4% women, 0.7% gender not specified) recruited online via the same social networking platforms used in Study 1. Participants were from the United States (60%), Canada (12%), Australia (19%), and the United Kingdom (9%). Participants ranged in age from 18 to 59 years (*M* = 25.55, *SD* = 6.43) and were all in a current romantic relationship (*M* = 3.55 years, *SD* = 4.27). Relationship status consisted of participants who were steadily dating (51%), cohabiting (25%), engaged (8%), and married (16%).

#### Materials and Procedure

This study received ethics approval from the Deakin University’s Human Ethics Committee. Participants completed an anonymous online survey which took approximately 20 min. The survey consisted of a series of demographic questions and self-report measures. These questions and measures are outlined below.

##### Demographics

Included general questions concerning the participant’s age, gender, country of residence, and relationship status and length.

##### Dehumanziation

Participants completed both the perpetration and target versions of DIIRS developed in Study 1 (see section “Study 1” for details).

##### Negative social exchanges

Negative relationship behaviors experienced within romantic relationships were assessed using the Test of Negative Social Exchanges (TENSE; Ruehlman and Karoly, 1991). The TENSE consists of 24 items that are rated on a nine-point Likert scale ranging from 1 (*not at all*) to 9 (*frequently*) where participants are asked to rate the frequency with which their relationship partner demonstrated negative behaviors. The TENSE is comprised of four subscales (six items per dimension): Hostility, Insensitivity, Interference, and Ridicule (αs > 0.79).

##### Caregiving

To assess the tendencies by which individuals render care to their romantic partner, participants completed the Caregiving Questionnaire (Kunce and Shaver, 1994). The Caregiving Questionnaire is a 32-item measure designed to assess four caregiving dimensions (eight items per dimension): proximity vs. distance, sensitivity vs. insensitivity, cooperation vs. control, and compulsive caregiving. Items are rated on a six-point Likert scale ranging from 1 (*totally disagree*) to 6 (*totally agree*; αs > 0.79).

##### Relationship quality

Participants’ appraisal of their current romantic relationship was assessed using the Perceived Relationship Quality Components short form (PRQC-SF; Fletcher et al., 2000). The PRQC-SF consists of six items that are rated on a seven-point scale ranging from 1 (*not at all*) to 7 (*extremely*; α = 0.83).

##### Communication patterns

Constructive and destructive communication patterns were assessed using the Communication Patterns Questionnaire short form (CPQ-SF; Furtis et al., 2010). The measure consists of 11 items comprising two subscales: demand/withdrawal (destructive communication), and positive communication (constructive communication). Items are rated on a nine-point Likert scale ranging from 1 (*very unlikely*) to 9 (*very likely*; αs > 0.72).

##### Positive regard

Positive appraisal of a romantic partner was assessed using a modified version of the Revised Barrett-Lennard Relationship Inventory (RBLR; Cramer, 1986). The RBLR consists of 16-items that are rated on a six-point Likert scale from 1 (*very untrue*) to 6 (*very true*; α = 0.90).

### Results and Discussion

Results are reported in two sections. In the first section, we report on the cross-validation of the construct validity of the two versions of the DIIRS using CFA. Specifically, we compared the structure of the best-fitting model identified in Study 1 [the four factor first-order model of dehumanization, Model 3 (Figure 2)] to all other factor structures illustrated across Models 1 to 5 (see Figures 1, 2). After estimating model fit, we conducted a series of model difference tests in which Model 3 was tested against all other models. In the second section, we report on the analyses pertaining to the criterion-related validity of the measure by way of correlational analyses in which the dimensions of both versions of the DIIRS were examined for their associations with the criterion-related variables.

#### Construct Validity

The evaluation of the four factor first-order structure of dehumanization (Model 3) demonstrated excellent fit to the data for both the perpetration version of the DIRRS and target version of the DIRRS (see Table 2). Consistent with the findings from Study 1, chi-square difference tests and ΔTLI revealed that the four factor first-order structure was significantly better in fit when compared to all other models [Δχ^2^ and ΔTLI pertain to Model 3 compared against Models 1, 2, 4, and 5 respectively (see Figures 1, 2)]. This was the case across both the perpetration and target versions of the DIRRS (see Table 2).

**TABLE 2:** Study 2 Chi-square difference tests for perpetration and target versions of the DIRRS.

**Model**	**χ^2^**	**df**	**CFI**	**TLI**	**RMSEA**	**SRMR**	**Comparison**	**χ^2^ Δ**	**df Δ**
**Perpetration**									
Model 1 (single factor)	1096.163	54	0.634	0.553	0.151	0.102	Model 1 and Model 3	867.622^∗∗∗^	6
Model 2 (2 correlated factors)	739.579	53	0.759	0.700	0.124	0.102	Model 2 and Model 3	511.038^∗∗∗^	5
Model 3 (4 correlated factors)	228.541	48	0.937	0.913	0.067	0.054	–	–	–
Model 4 (4 first-order factors, 2 second-order factors)	232.754	49	0.936	0.923	0.067	0.056	Model 4 and Model 3	4.213^∗^	1
Model 5 (4 first-order factors, 1 second-order factor)	243.693	50	0.932	0.910	0.068	0.056	Model 5 and Model 3	15.152^∗∗^	2
**Target**									
Model 1 (single factor)	1278.656	54	0.665	0.590	0.164	0.102	Model 1 and Model 3	954.969^∗∗∗^	6
Model 2 (2 correlated factors)	1015.654	53	0.736	0.672	0.147	0.085	Model 2 and Model 3	691.967^∗∗∗^	5
Model 3 (4 correlated factors)	323.687	48	0.925	0.900	0.080	0.053	–	–	–
Model 4 (4 first-order factors, 2 second-order factors)	331.658	49	0.923	0.896	0.083	0.057	Model 4 and Model 3	0.038^∗∗^	1
Model 5 (4 first-order factors, 1 second-order factor)	333.704	50	0.922	0.897	0.082	0.055	Model 5 and Model 3	0.125^∗∗^	2

*χ^2^, Chi-square; df, degrees of freedom; CFI, Comparative Fit Index; TLI, Tucker Lewis Index; RMSEA, root mean square error of approximation; SRMR, standardized root mean residual. ^∗^*p* < 0.05, ^∗∗^*p* < 0.01, ^∗∗∗^*p* < 0.001.*

The findings regarding construct validity for Study 2, replicate the findings of Study 1, such that the four factor first-order solution was the best fit to the data. Importantly, in Study 2, these findings were consistent for models testing items from both perpetrator and target perspectives. These findings again suggest that when it comes to interpersonal dehumanization—such as within romantic relationships—dehumanization may be more nuanced than first thought. This is not to say that the dual model (Haslam, 2006) or unitary conceptualizations of dehumanization are not relevant to the study of dehumanization within romantic relationships. Rather, the one and two factor higher-order models reflect broad dimensions that are best distilled into more fine-grained facets within the context of romantic relationships. Importantly, these related but distinct facets capture a number of primary themes that are often implicitly discussed in reference to human uniqueness and human nature, but not readily identified as part of the one or two factor approaches.

With reference to the four factor first-order structure of the DIRRS, while the subfactors of immature and unrefined both reflect denials of human uniqueness, they are also conceptually distinct. For example, an individual may perceive their partner to be highly competent and mature, but nonetheless lack a degree of social refinement. Likewise, although the exploitable and emotionless subfactors reflect denials of human nature, treating someone with conditional regard (i.e., as if their value lies only in what they can offer their partner) is not synonymous with treating someone as if they are heartless or unable to experience or express interpersonal warmth (i.e., emotionless). Thus, an individual can possess interpersonal warmth and emotionality while at the same time be treated as if they are only valuable for what they can offer. In contrast, the emotionless subfactor reflects a constant state of emotional inertia (i.e., being heartless and cold).

#### Criterion Validity

Based on the results of the construct validity analyses, we evaluated the criterion-related validity of the four factor first-order structure of the perpetrator and target versions of the DIRRS. Specifically, the associations between the four factors of immature, unrefined, exploitable, and emotionless, and variables representing positive and negative relationship interactions as well as relationship and partner evaluations were tested.

The descriptive statistics and zero-order correlations between the four factors of the DIRRS (for both perpetration and target versions) and the criterion-related validity variables are listed in Tables 3, 4. In line with predictions, for both versions of the DIRRS, the four subscales were found to positively correlate with the use of demand-withdrawal communication (i.e., destructive conflict management) and negatively with positive communication (i.e., constructive conflict management). Similarly, across both versions of the measure, all four subscales were negatively associated with reports of relationship quality and regard for one’s partner (see Tables 3, 4). Additionally, moderate associations were found between all subscales of the target version of the DIRRS and scores on negative relationship experiences as indexed by the TENSE subscales of hostility, insensitivity, interference, and ridicule (see Table 3). Finally, and in line with our predictions, all subscales of the perpetration version of the DIRRS were positively associated with providing care that is compulsive and intrusive, and negatively associated with care that is sensitive, proximal, and cooperative (see Table 4).

**TABLE 3:** Study 2 descriptive statistics and zero-order correlations between the target version of the DIRRS and other study variables.

	**1**	**2**	**3**	**4**	**5**	**6**	**7**	**8**	**9**	**10**	**11**	**12**
(1) Immature	–											
(2) Unrefined	0.43^∗∗^	–										
(3) Exploitable	0.40^∗∗^	0.57^∗∗^	–									
(4) Emotionless	0.36^∗∗^	0.38^∗∗^	0.45^∗∗^	–								
(5) Hostility	0.49^∗∗^	0.37^∗∗^	0.44^∗∗^	0.42^∗∗^	–							
(6) Insensitivity	0.44^∗∗^	0.47^∗∗^	0.65^∗∗^	0.38^∗∗^	0.69^∗∗^	–						
(7) Interference	0.49^∗∗^	0.42^∗∗^	0.59^∗∗^	0.50^∗∗^	0.69^∗∗^	0.66^∗∗^	–					
(8) Ridicule	0.49^∗∗^	0.55^∗∗^	0.62^∗∗^	0.50^∗∗^	0.68^∗∗^	0.82^∗∗^	0.64^∗∗^	–				
(9) Relationship quality	–0.29^∗∗^	–0.31^∗∗^	–0.48^∗∗^	–0.34^∗∗^	–0.32^∗∗^	–0.44^∗∗^	–0.39^∗∗^	–0.46^∗∗^	–			
(10) Demand-withdrawal	0.38^∗∗^	0.35^∗∗^	0.40^∗∗^	0.39^∗∗^	0.51^∗∗^	0.56^∗∗^	0.50^∗∗^	0.48^∗∗^	–0.32^∗∗^	–		
(11) Positive communication	–0.28^∗∗^	–0.31^∗∗^	–0.43^∗∗^	–0.28^∗∗^	–0.24^∗∗^	–0.41^∗∗^	–0.31^∗∗^	–0.41^∗∗^	0.51^∗∗^	–0.45^∗∗^	–	
(12) Positive regard	–0.35^∗∗^	–0.29^∗∗^	–0.50^∗∗^	–0.46^∗∗^	–0.41^∗∗^	–0.44^∗∗^	–0.51^∗∗^	–0.45^∗∗^	0.72^∗∗^	–0.42^∗∗^	0.47^∗∗^	–
Mean Standard deviation *N* 847	2.12	1.52	1.67	1.82	2.34	2.61	2.13	2.21	5.80	18.64	19.65	5.40
	1.03	0.77	0.88	2.34	1.46	1.65	1.21	1.30	0.98	8.74	5.43	0.63

*^∗∗^*p* < 0.01.*

**TABLE 4:** Study 2 descriptive statistics and zero-order correlations between the perpetration version of the DIRRS and other study variables.

	**1**	**2**	**3**	**4**	**5**	**6**	**7**	**8**	**9**	**10**		
(1) Immature	–											
(2) Unrefined	0.48^∗∗^	–										
(3) Exploitable	0.38^∗∗^	0.45^∗∗^	–									
(4) Emotionless	0.27^∗∗^	0.27^∗∗^	0.33^∗∗^	–								
(5) Proximal caregiving	–0.27^∗∗^	–0.34^∗∗^	–0.37^∗∗^	–0.12^∗∗^	–							
(6) Sensitive caregiving	–0.19^∗∗^	–0.25^∗∗^	–0.33^∗∗^	–0.22^∗∗^	0.42^∗∗^	–						
(7) Cooperative caregiving	–0.51^∗∗^	–0.36^∗∗^	–0.34^∗∗^	–0.20^∗∗^	0.33^∗∗^	0.37^∗∗^	–					
(8) Compulsive caregiving	0.28^∗∗^	0.17^∗^	0.10^∗∗^	0.19^∗∗^	0.03	–0.15^∗∗^	–0.43^∗∗^	–				
(9) Relationship quality	–0.33^∗∗^	0.35^∗∗^	–0.40^∗∗^	–0.35^∗∗^	0.43^∗∗^	0.36^∗∗^	–0.25^∗∗^	0.05	–			
(10) Positive regard	–0.48^∗∗^	–0.53^∗∗^	–0.51^∗∗^	–0.35^∗∗^	0.57^∗∗^	0.38^∗∗^	–0.42^∗∗^	0.10^∗∗^	0.72^∗∗^	–		
(11) Demand-withdrawal	0.43^∗∗^	0.31^∗∗^	0.34^∗∗^	0.36^∗∗^	–0.27^∗∗^	–0.31^∗∗^	–0.44^∗∗^	–0.28^∗∗^	–0.32^∗∗^	–0.42^∗∗^	–	
(12) Positive communication	–0.26^∗∗^	−0.24^∗^	–0.31^∗∗^	–0.31^∗∗^	0.30^∗∗^	0.37^∗∗^	0.28^∗∗^	0.15^∗^	0.51^∗∗^	0.47^∗∗^	–0.45^∗∗^	–
Mean Standard deviation *N* 847	2.25	1.53	1.45	1.70	5.31	4.46	4.37	3.08	5.80	5.40	18.64	19.65
	1.11	0.75	0.65	0.95	0.72	0.94	0.90	0.96	0.98	0.63	8.74	5.43

*^∗^*p* < 0.05, ^∗∗^*p* < 0.01.*

In summary, being the target or perpetrator of dehumanization has implications for the way romantic partners treat one another and their appraisals of their partner and relationship. A consistent pattern of negative associations with positive relationship behaviors, and positive associations with negative relationship behaviors was found between all dehumanization subscales (for both target and perpetration versions of the DIRRS). In short, both versions of the DIRRS and their respective factors were associated with criterion-related variables in ways that were in line with our predictions.

## Study 3

Study 3 had two aims. First, to cross-validate the associations between dehumanization and the criterion-validity variables reported in Study 2. Second, to investigate the associations between dehumanization and the very extreme end of the negative relationship behavior spectrum—overt emotional and physical abuse.

The application of dehumanization to the study of more severe romantic relationship behavior stems from the long-held view that perpetrators of dehumanization are less likely to experience empathic distress or guilt for an abhorrent or violent act and are less likely to condemn oneself for such an act (e.g., Kelman, 1976; Bandura, 1999; Haslam and Loughnan, 2014; Kteily and Bruneau, 2017). Because the perpetrator perceives the target of dehumanization to be someone who is less than human (and thus not worthy of moral concern or respectful treatment; Haslam, 2015) it stands to reason that within romantic relationships, a partner that dehumanizes their significant other may also engage in emotional abuse, domestic violence, and other abusive maltreatments.

Furthermore, based on previous findings in which dehumanization has been shown to have a negative impact on self-perception (e.g., feelings of shame and guilt; Bastian and Haslam, 2011), we also examined the association between dehumanization and positive appraisals of the self (i.e., feeling proud, confident, and strong).

Consistent with our predictions in Study 2, we expected that being the target and perpetrator of dehumanization would be negatively associated with positive relationship behaviors, positively associated with negative relationship behaviors (including emotional and physical abuse), and negatively associated with relationship quality. It was also predicted that being the target of dehumanization would be negatively associated with positive self-appraisals.

### Method

#### Participants

The sample consisted of 328 participants (27% men, 72% women, 1% gender not specified) recruited online (using the same social networking sites as Studies 1 and 2) from the United States (51%), Canada (5%), Australia (42%), and New Zealand (2%). Participants ranged in age from 18 to 60 years (*M* = 23.40, *SD* = 6.43) and were all currently in a romantic relationship (*M* = 2.40 years, *SD* = 4.27). Relationship status consisted of participants who were steadily dating (51%), cohabiting (25%), engaged (8%), and married (16%).

#### Materials and Procedure

This study received ethics approval from the Deakin University’s Human Ethics Committee. As with Studies 1 and 2, participants completed an anonymous online survey. Participation took approximately 20 min. The survey contained many of the same assessments used in Study 2 [i.e., the same demographic questions, the DIRRS to assess being the target and perpetrator of dehumanization, the TENSE (Ruehlman and Karoly, 1991) to assess negative interactions, the CPQ-SF (Furtis et al., 2010) to assess communication patterns, and the PRQC-SF (Fletcher et al., 2000)] to measure relationship quality. In addition to these measures, the survey included measures of emotional and physical abuse and self-appraisals. These are described below.

##### Emotional abuse

Participants completed the restrictive engulfment (seven-items) and dominance/intimidation (seven-items) subscales of the Multidimensional Measure of Emotional Abuse (Murphy and Hoover, 1999) in order to provide an assessment of both their experience and perpetration of emotional abuse. Participants are required to rate emotional abuse items in terms of the number of times they and their partner enacted the abuse in the past 6-months [i.e., 0 (*never in the past 6 months*) to 6 (>*20 times)*]. Items were summed to create a total emotional abuse experience score (α = 0.80) and a total emotional abuse perpetration score (α = 0.75) for each participant.

##### Physical abuse

Participants also completed the overt physical violence (seven-items) and restrictive violence (three-items) subscales of the Abuse Within Intimate Relationships Scale (AIRS; Borjesson et al., 2003). Participants were asked to indicate how often physical abuse behaviors occur in their current romantic relationship on a seven-point rating scale ranging from 1 (*never*) to 7 (*always*). Items were asked twice, once to assess the experience of physical abuse (α = 0.80), and again to assess the perpetration of physical abuse (α = 0.90).

##### Self-appraisal

Participants were required to rate the extent to which they generally feel strong, proud, and confident. Each of these items is rated on a five-point scale ranging from 1 (*very slightly, or not at all*) to 5 (*extremely*). Scores from these items were then averaged to create a composite score of positive self-appraisal (α = 0.73).

### Results and Discussion

The descriptive statistics and zero-order correlations between the four factors of the DIRRS (for both perpetration and target versions) and the criterion-related validity variables are presented in Tables 5, 6. As predicted (and consistent with Study 2 results), across both versions of the DIRRS, the four subscales were found to positively correlate with the use of demand-withdrawal communication and negatively with positive communication. Similarly, across both versions of the measure, all four subscales were negatively associated with reports of relationship quality (see Tables 5, 6). Additionally, for the target version of the DIRRS, moderate associations were again found (see section “Study 2”) between the dehumanization subscales and reports of partner hostility, insensitivity, interference, and ridicule (as indexed by the TENSE subscales; see Table 5).

**TABLE 5:** Study 3 descriptive statistics and zero-order correlations between the perpetration version of the DIRRS and other study variables.

	**1**	**2**	**3**	**4**	**5**	**6**	**7**	**8**	**9**
(1) Immature	–								
(2) Unrefined	0.46^∗∗^	–							
(3) Exploitable	0.37^∗∗^	0.37^∗∗^	–						
(4) Emotionless	0.31^∗∗^	0.15^∗^	0.42^∗∗^	–					
(5) Relationship quality	–0.35^∗∗^	–0.32^∗∗^	−0.34^∗^	–0.33^∗∗^	–				
(6) Emotional abuse perpetration	0.32^∗∗^	0.22^∗∗^	0.24^∗∗^	0.38^∗∗^	–0.26^∗∗^	–			
(7) Physical abuse perpetration	0.30^∗∗^	0.12^∗^	0.30^∗∗^	0.29^∗∗^	–0.23^∗∗^	0.18^∗∗^	–		
(8) Demand-withdrawal	0.47^∗∗^	0.32^∗∗^	0.41^∗∗^	0.50^∗∗^	–0.27^∗∗^	0.46^∗∗^	0.24^∗∗^	–	
(9) Positive communication	–0.33^∗∗^	−0.13^∗^	–0.26^∗∗^	–0.36^∗∗^	0.35^∗∗^	–0.24^∗∗^	–0.21^∗∗^	–0.46^∗∗^	–
Mean Standard deviation *N* 328	2.01	1.31	1.34	1.68	6.09	4.57	1.10	21.16	6.09
	1.03	0.61	0.58	1.01	0.80	5.78	0.31	6.09	0.80

*^∗^*p* < 0.05, ^∗∗^*p* < 0.01.*

**TABLE 6:** Study 3 descriptive statistics and zero-order correlations between the target version of the DIRRS and other study variables.

	**1**	**2**	**3**	**4**	**5**	**6**	**7**	**8**	**9**	**10**	**11**	**12**	**13**	**14**
(1) Immature	–													
(2) Unrefined	0.48^∗∗^	–												
(3) Exploitable	0.48^∗∗^	0.47^∗∗^	–											
(4) Emotionless	0.47^∗∗^	0.29^∗∗^	0.43^∗∗^	–										
(5) Hostility	0.56^∗∗^	0.44^∗∗^	0.51^∗∗^	0.51^∗∗^	–									
(6) Insensitivity	0.41^∗∗^	0.45^∗∗^	0.66^∗∗^	0.34^∗∗^	0.59^∗∗^	–								
(7) Interference	0.27^∗∗^	0.27^∗∗^	0.32^∗∗^	0.46^∗∗^	0.43^∗∗^	0.38^∗∗^	–							
(8) Ridicule	0.24^∗∗^	0.28^∗∗^	0.20^∗∗^	0.18^∗∗^	0.26^∗∗^	0.15^∗∗^	0.23^∗∗^	–						
(9) Relationship quality	–0.26^∗∗^	–0.29^∗∗^	–0.42^∗∗^	–0.44^∗∗^	–0.31^∗∗^	–0.45^∗∗^	–0.42^∗∗^	–0.11	–					
(10) Demand-withdrawal	0.44^∗∗^	0.30^∗∗^	0.46^∗∗^	0.49^∗∗^	0.60^∗∗^	0.49^∗∗^	0.30^∗∗^	0.25^∗∗^	–0.27^∗∗^	–				
(11) Positive communication	–0.34^∗∗^	–0.31^∗∗^	–0.37^∗∗^	–0.24^∗∗^	–0.36^∗∗^	–0.39^∗∗^	–0.21^∗∗^	–0.11	0.35^∗∗^	–0.46^∗∗^	–			
(12) Emotional abuse target	0.40^∗∗^	0.40^∗∗^	0.40^∗∗^	0.53^∗∗^	0.60^∗∗^	0.40^∗∗^	0.48^∗∗^	0.30^∗∗^	–0.34^∗∗^	0.46^∗∗^	–0.30^∗∗^	–		
(13) Physical abuse target	0.12^∗^	0.06	0.19^∗∗^	0.17^∗∗^	0.15^∗∗^	0.20^∗∗^	0.12^∗^	0.15^∗∗^	–0.16^∗∗^	0.25^∗∗^	–0.08	0.21^∗∗^	–	
(14) Self-appraisal	−0.14^∗^	–0.09	−0.15^∗^	−0.21^∗^	–0.07	−0.14^∗^	–0.10	–0.03	0.23^∗^	−0.14^∗^	0.19^∗∗^	−0.13^∗^	0.02	–
Mean Standard deviation *N* 328	2.04	1.41	1.51	1.66	2.00	1.72	1.50	1.39	18.46	21.16	6.09	4.15	1.10	3.32
	1.19	0.81	0.85	1.07	0.66	0.75	0.63	0.59	9.00	6.09	0.80	6.30	0.23	0.89

*^∗^*p* < 0.05, ^∗∗^*p* < 0.01.*

In line with further predictions, all dehumanization subscales of the DIRRS were moderately positively correlated with the experience of emotional abuse (see Tables 5, 6). In addition, scores on all but one DIRRS subscale [i.e., unrefined (target version)] were significantly positively correlated with the experience of physical abuse. Similarly, scores on all but the unrefined subscale of the target version of the DIRRS were found to correlate negatively with positive self-appraisals. While the unrefined subscale for the target version of the DIRRS failed to reach significance for both the physical abuse and self-appraisal associations, the direction of these correlations was consistent with the other dehumanization factors.

In summary, the results of Study 3 provide support for the criterion validity findings from Study 2 for both the target and perpetration versions of the DIRRS. The reported associations between the target dehumanization subscales and self-appraisals are also consistent with previous research in which dehumanization has been shown to have a negative association with self-perception (e.g., Bastian and Haslam, 2011). In particular, being denied humanness by one’s romantic partner appears to have implications for how individuals perceive themselves in terms of variables related to ego and self-efficacy (i.e., feeling proud, confident, and strong). Furthermore, the findings of the current study suggest that dehumanization is associated with extreme and serious negative relationship behaviors, namely emotional and physical abuse. Findings from Study 3, therefore, further support the link between dehumanization and the unacceptable treatment of others. This suggests that within romantic relationships denying humanness may give people a license to aggress or abuse their partner.

## General Discussion

Despite increasing interest in the study of interpersonal dehumanization, there remains little research into this phenomenon within the context of romantic relationships. A major barrier in linking dehumanization to the study of romantic relationships is that no measure exists to assess dehumanization within this interpersonal context. Furthermore, ambiguity exists regarding the factor structure of interpersonal dehumanization. With these limitations in mind, the current paper reports on the development and psychometric evaluation of the Dehumanization in Romantic Relationships Scale (DIRRS). The DIRRS significantly extends on past measures of dehumanization by providing a multifaceted assessment of dehumanization within romantic relationships that accounts for both perpetrator and target perspectives.

The current paper advances on current conceptualizations of dehumanization by proposing that dehumanization within romantic relationships (and possibly, interpersonal relationships more generally) consists of four related but distinct facets (each possessing good internal reliability). This novel factor structure decomposes the denial of human uniqueness and human nature into two factors respectively—immature and unrefined (denials of human uniqueness), and exploitable and emotionless (denials of human nature). Not only do these facets represent key features of the denial of humanness described in the literature (e.g., Haslam, 2006, 2015; Bastian and Haslam, 2011; Haslam et al., 2013), but the implication is that not all denials of human uniqueness should be considered as conceptually equivalent; likewise, for denials of human nature. That is, to treat a partner as immature or incompetent is not the same as treating them as lacking social refinement. Similarly, treating someone with conditional regard is not analogous to engaging in dissociation. The factor structure constituting these facets was replicated across Studies 1 and 2, with the four factor first-order model consistently found to be of better fit compared to all other factor structures. Moreover, the same four factor model equally applied to the perpetrator and target versions of the DIRRS.

Across Studies 2 and 3, the dehumanization facets in both versions of the DIRRS were also associated with criterion-related variables in ways that were in line with predictions. All four facets were positively associated with destructive communication patterns, negative partner behaviors such as hostility, insensitivity, interference (including care that was intrusive), and ridicule. In contrast the facets were negatively associated with constructive communication patterns, the provision of sensitive-proximal care, relationship quality, regard for one’s partner and positive self-appraisals (only measured in Study 3). These findings provide the first evidence that romantic relationship dehumanization attenuates positive relationship appraisals and functioning but amplifies negative appraisals of oneself and partner, exacerbating negative relationship processes. Furthermore, the associations found between the DIRRS and emotional and physical abuse (Study 3) highlight that dehumanization is indeed associated with relationship behaviors that extend into the realm of intimate partner violence and aggression. In this way, the findings of Study 3 suggest that just as dehumanization may be an explanatory mechanism for intergroup prejudice, discrimination and aggression (e.g., Bandura, 1999; Haslam, 2015; Kteily et al., 2015; Kteily and Bruneau, 2017), so too, may dehumanization act as an impelling factor in the manifestation of interpersonal abuse. Therefore, future research should investigate dehumanization as a possible explanatory mechanism for the manifestation of abuse within romantic relationships.

Although the four factor first-order model of dehumanization (see Figure 2, Model 3) was the best fitting model (see Tables 1, 2), we note that both the one and two factor higher-order models (see Figure 2, Models 4 and 5) also produced good to very good fit. This suggests that the four first-order factors may indeed represent specific substrates of human uniqueness, human nature, or dehumanization overall. Thus, researchers may also be interested in using the DIRRS to compute higher-order factors^3^ if they wish to aggregate up to more global assessments of interpersonal dehumanization. It is important to note, however, that omitting the four factors from the modeling of dehumanization by way of the DIRRS reduces fit significantly—which speaks to the substantive and empirical need to model these dehumanization facets within the context of romantic relationships.

### Limitations and Future Directions

Although we find highly consistent results across the three studies in terms of the construct and criterion-related validity of the DIRRS, each sample was by-and-large a community, non-clinical sample that on average reported low levels of dehumanization. Researchers, therefore, should attempt to confirm our findings using a more diverse sample including distressed or high-conflict couples. An understanding of how couples characterized by high stress and negativity engage in dehumanization may provide important insights into understanding key contextual factors affecting couples and families. These factors may include financial stress, a history of troublesome family relationships, and other contextual variables indicative of harsh or unpredictable environments. In addition, although the four dehumanization factors are identified statistically, and on strong theoretical grounds, the criterion validation performed on the DIRRS does not demonstrate whether the various factors predict different behaviors within relationships (i.e., we did not differentiate between each factor’s predictive validity). Furthermore, future research should implement the DIRRS within dyadic contexts, in order to account for the influence that both members of a couple have on their own and their partner’s experience of being the perpetrator and target of dehumanization.

In addition, although we developed and validated the DIRRS using data from individuals currently in romantic relationships, we believe the measure can be readily adapted to assess other types of relationships, including parent-child relationships and relationships between peers and colleagues. Finally, future research should investigate whether dehumanization plays a causal role in the enactment of emotional and physical abuse, or merely a variable that is associated with, but not a causal factor of, negative relationship experiences.

## Conclusion

Despite increasing scholarly interest in the concept of dehumanization, and recent application of the phenomenon to the study of interpersonal processes, we still know very little about the manifestation of dehumanization within romantic relationships. We also do not clearly understand the consequences of being denied humanness by one’s romantic partner. This paper has made some novel and important contributions to the study of dehumanization, enhancing the field’s understanding of how dehumanization is conceptualized, assessed, and evaluated within romantic relationships. It is hoped that the DIRRS spurs on dehumanization research within the context of romantic (and other close) relationships, continuing the growth of dehumanization research within the field of social psychology, and in particular the study of interpersonal processes.

## Data Availability Statement

The datasets generated for this study are available on request to the corresponding author.

## Ethics Statement

The studies involving human participants were reviewed and approved by the Deakin University’s Human Ethics Committee. The patients/participants provided their written informed consent to participate in this study.

## Author Contributions

BP and GK conceived the presented idea, carried out the study, and performed the analytical computations. EM provided critical feedback and helped to shape the research. All authors contributed to the final manuscript.

## Conflict of Interest

The authors declare that the research was conducted in the absence of any commercial or financial relationships that could be construed as a potential conflict of interest.

## Footnotes

1 Bastian and colleagues focused on the self-dehumanizing consequences of social ostracism and in doing so directly measured participants’ perceptions of their self-humanity after a dehumanizing experience.

2 After reading a vignette in which they were the target of a particular maltreatment, participants were required to rate the extent to which the perpetrator viewed them as fully human (e.g., “the other person doesn’t see me as an individual”).

3 In a series of preliminary analyses, we calculated human nature, human uniqueness and dehumanization overall subscales to represent the one and two factor higher-order models, respectively. Associations between these broader subscales and previously mentioned criterion-related variables were found to be similar to what is noted in Tables 1–6.

## Appendix A

The Dehumanization in Romantic Relationships Scale (DIRRS) consists of 24 items (12 items that assess dehumanization perpetration and 12 items that assess dehumanization receipt). Each statement is answered on a seven-point Likert scale (ranging from 1 = *strongly disagree* to 7 = *strongly agree*). For the perpetration items, instructions are to rate each item based on how the individual generally behaves in their romantic relationship. For the target items, instructions are to rate each item based on how their romantic partner generally behaves. Factor categories are shown below as subheadings and should be omitted when the scale is administered.

**Table:**

Generally, I treat my partner…	Generally, my partner treats me…
*Immature*	*Immature*
… as if they were a child	… as if I am a child^∗^
… as if they are immature	… as if I am immature^∗^
… as if they can’t manage on their own	… as if I can’t manage on my own
*Unrefined*	*Unrefined*
… as if they embarrass me	… as if I embarrass them
… as if they lack social status	… as if I lack social status
… as if I am ashamed of them	… as if they are ashamed of me
*Exploitable*	*Exploitable*
… as a means to an end	… as a means to an end^∗^
… as if they are only valuable for what they can offer	… as if I am only valuable for what I can offer
… as if their opinion doesn’t count	… as if my opinion doesn’t count
*Emotionless*	*Emotionless*
… as if they are heartless	… as if I am heartless
… as if they are unresponsive	… as if I am unresponsive
… as if they are cold	… as if I am cold

*^∗^Denotes items retained from Bastian and Haslam (2011) measure of dehumanization.*
